# Forward-viewing echoendoscope plus gel immersion technique in a patient with Billroth II reconstruction

**DOI:** 10.1055/a-1841-6048

**Published:** 2022-06-03

**Authors:** Tatsuya Ishii, Tsuyoshi Hayashi, Kuniyuki Takahashi, Toshifumi Kin, Akio Katanuma

**Affiliations:** Center for Gastroenterology, Teine Keijinkai Hospital, Sapporo, Hokkaido, Japan

Endoscopic ultrasound (EUS) in patients with surgically altered anatomy is often difficult, and forward-view echoendoscopy (FV-EUS) has proven to be useful in such cases 1 . Recently, the gel immersion technique has been used for various endoscopic diagnostic and therapeutic procedures 2 3 4 . In the case reported here, the bile ducts and papilla were successfully observed in a patient with a Billroth II reconstruction using FV-EUS and the gel immersion technique ( Video 1 ).

**Video 1** The intrapancreatic pancreaticobiliary ducts and papilla were successfully observed in a patient with Billroth II reconstruction using forward-view echoendoscopy and the gel immersion technique.


An 82-year-old man who had undergone distal gastrectomy with Billroth II reconstruction for a gastric ulcer was referred to our center with elevated hepatobiliary enzymes and suspected cholangitis. Blood tests had not revealed an identifiable cause of hepatitis, while abdominal ultrasonography, computed tomography, and magnetic resonance cholangiopancreatography showed no bile duct dilatation and no obvious origin of the obstruction ( Fig. 1 ).

**Fig. 1:**
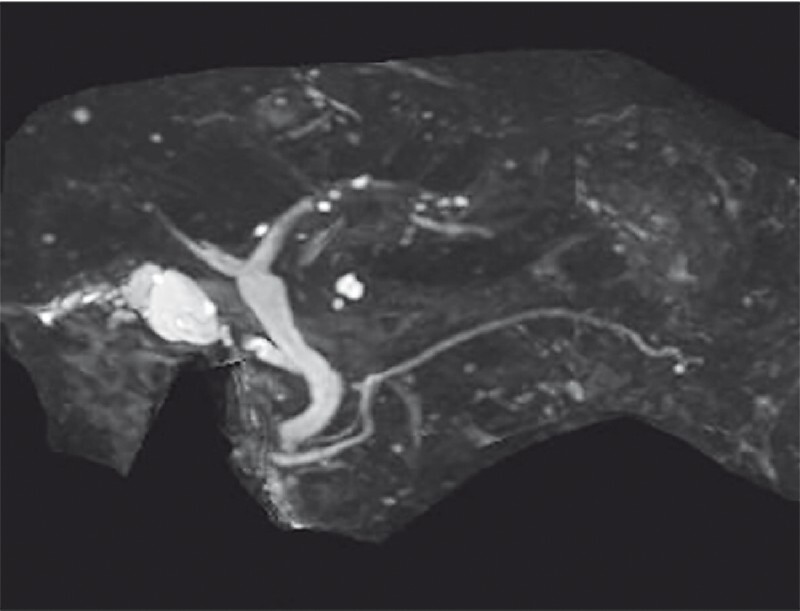
Magnetic resonance cholangiopancreatography demonstrated no apparent obstruction of the bile ducts.

EUS was performed using a linear echoendoscope; however, scope insertion into the afferent limb was difficult and the bile ducts could not be visualized in the stomach region. By switching to FV-EUS (TGF-UC260J; Olympus Medical Systems Corp., Tokyo, Japan), we safely guided the scope to the afferent limb with visualization of the anastomosis and successfully reached the ampulla of Vater. FV-EUS revealed mild dilatation of the common bile duct, with no obvious stones. The peripapillary delineation was unclear, making it difficult to detect the presence of small stones or tumors ( Fig. 2 ). For gel immersion, the duodenum was filled with gel (VISCOCLEAR; Otsuka Pharmaceutical Factory, Tokushima, Japan) via the scope working channel. The intrapancreatic pancreaticobiliary ducts and papilla could then be delineated clearly ( Fig. 3 ). Therefore, with the absence of stones/tumors confirmed, endoscopic retrograde cholangiopancreatography was deemed unnecessary. Thereafter, the patient’s hepatobiliary enzyme levels slowly decreased. In the afferent limb, the gel tended to pool at the blind end and could be clearly observed.

**Fig. 2:**
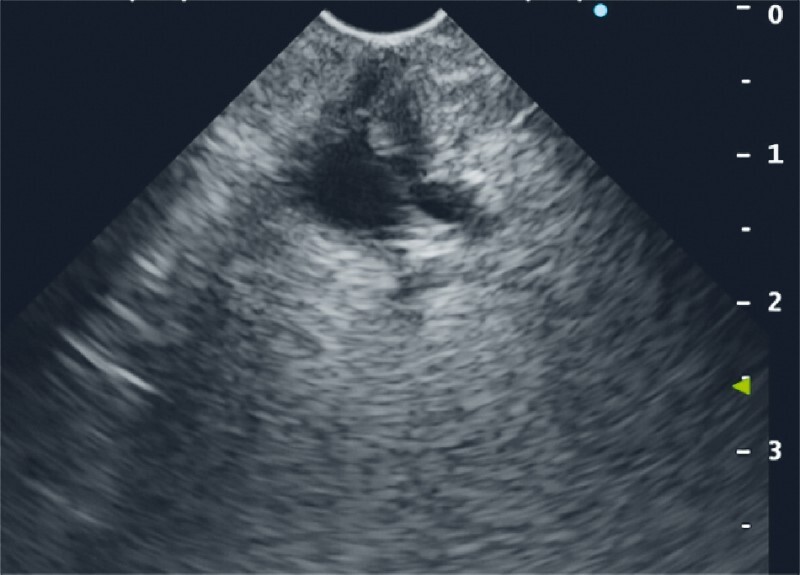
View during forward-view echoendoscopy showing that, although visualization of the intrapancreatic bile ducts and papilla was possible, detailed evaluation was difficult.

**Fig. 3:**
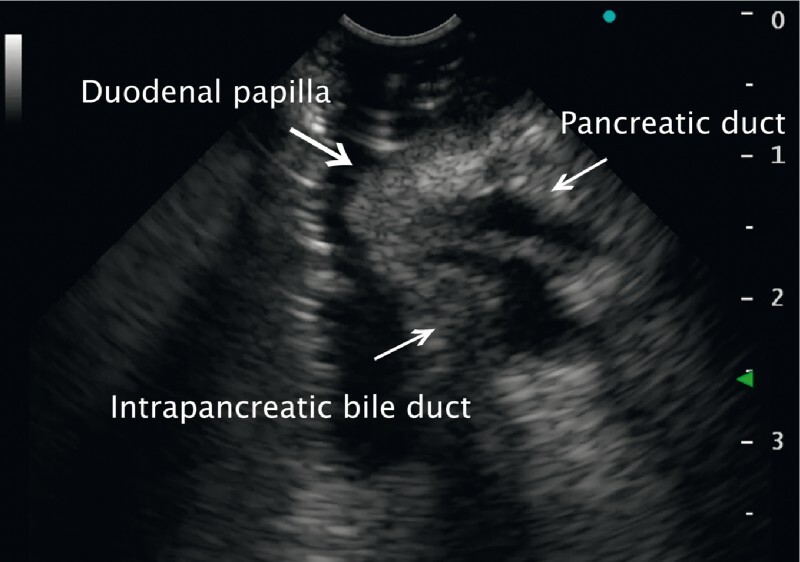
Gel immersion endoscopic ultrasound view clearly showing the papilla and intrapancreatic bile duct.

FV-EUS plus gel immersion was considered to be the best combination for pancreaticobiliary examination in such patients with surgically altered anatomy.

Endoscopy_UCTN_Code_CCL_1AF_2AF

## Notes

Endoscopy E-Videos

https://eref.thieme.de/e-videos
*Endoscopy E-Videos* is an open access online section, reporting on interesting cases and new techniques in gastroenterological endoscopy. All papers include a high quality video and all contributions are freely accessible online. Processing charges apply (currently EUR 375), discounts and wavers acc. to HINARI are available. This section has its own submission website at https://mc.manuscriptcentral.com/e-videos

## References

[1] Fusaroli P Serrani M Lisotti A Performance of the forward-view echoendoscope for pancreaticobiliary examination in patients with status post-upper gastrointestinal surgery Endosc Ultrasound 2015 4 336 341 26643703 10.4103/2303-9027.170427 PMC4672593

[2] Yano T Nemoto D Ono K Gel immersion endoscopy: a novel method to secure the visual field during endoscopy in bleeding patients (with videos) Gastrointest Endosc 2016 83 809 811 26463338 10.1016/j.gie.2015.09.048

[3] Hanaoka N Ishihara R Matsuura N Esophageal EUS by filling water-soluble lubricating jelly for diagnosis of depth of invasion in superficial esophageal cancer Gastrointest Endosc 2015 82 164 165 25922256 10.1016/j.gie.2015.01.028

[4] Toyonaga H Takahashi K Kin T Gel immersion technique for the examination and treatment of an ampullary tumor Endoscopy 2022 54 E115 E116 33784756 10.1055/a-1408-0458

